# Supplementary material for: Complexity of the relationship between life expectancy and overlap of lifespans
Source: PLoS One. 2018 Jul 12;13(7):e0197985. doi: 10.1371/journal.pone.0197985 (PMC6042694; doi:10.1371/journal.pone.0197985)
Supplement: S1 Appendix — (PDF) [file pone.0197985.s001.pdf]

S1 Appendix

Life expectancy at any age x $e(x)$, where the hazard of death μ is constant over age and $l(0) = 1$, is

$$e(x) = \frac{1}{l(x)} \int_x^\omega l(0) l(t) \mathrm{d}t \tag{1}$$
$$= \frac{1}{l(x)} \int_x^\omega l(0) e^{-\mu t} \mathrm{d}t \tag{2}$$
$$= \frac{1}{l(0)e^{-\mu x}} \left[\frac{e^{-\mu t}}{-\mu} \right]_x^\omega \tag{3}$$
$$= \frac{1}{e^{-\mu x}} \left(\frac{e^{-\mu x}}{\mu} - \frac{e^{-\mu \omega}}{\mu} \right) \tag{4}$$
$$= \frac{1}{\mu}, \tag{5}$$

with $\lim_{\omega \to \infty} e^{-\mu\omega} = 0$.

The shared life expectancy at any age x $\tau(x)$, where the hazard of death μ is constant over age and $l(0) = 1$, is

$$\tau(x) = \frac{1}{l(x)^2} \int_x^\omega l(t)^2 \mathrm{d}t \tag{6}$$
$$= \frac{1}{l(x)^2} \int_x^\omega \left(e^{-\mu t} \right)^2 \mathrm{d}t \tag{7}$$
$$= \frac{1}{l(x)^2} \int_x^\omega l(0) e^{-\mu t} l(0) e^{-\mu t} \mathrm{d}t \tag{8}$$
$$= \frac{1}{l(x)^2} \int_x^\omega l(0)^2 e^{-2\mu t} \mathrm{d}t \tag{9}$$
$$= \frac{1}{l(0)^2 e^{-2\mu x}} \left[\frac{e^{-2\mu t}}{-2\mu} \right]_x^\omega \tag{10}$$
$$= \frac{1}{e^{-2\mu x}} \left(\frac{e^{-2\mu x}}{2\mu} - \frac{e^{-2\mu\omega}}{2\mu} \right) \tag{11}$$
$$= \frac{1}{2\mu}, \tag{12}$$

with $\lim_{\omega \to \infty} e^{-2\mu\omega} = 0$.

The proportion of life shared from birth $\bar{l}_0$, when the hazard of death μ is constant over age is therefore

$$\bar{l}_0 = \frac{\tau_0}{e_0} = \frac{\frac{1}{2\mu}}{\frac{1}{\mu}} = \frac{1}{2}. \tag{13}$$
